# Seasonal and Sexual Differences in the Microbiota of the Hoopoe Uropygial Secretion

**DOI:** 10.3390/genes9080407

**Published:** 2018-08-11

**Authors:** Sonia M. Rodríguez-Ruano, Manuel Martín-Vivaldi, Juan M. Peralta-Sánchez, Ana B. García-Martín, Ángela Martínez-García, Juan J. Soler, Eva Valdivia, Manuel Martínez-Bueno

**Affiliations:** 1 Departamento de Microbiología, Universidad de Granada, E-18071 Granada, Spain; jmps@ugr.es (J.M.P.-S.); evavm@ugr.es (E.V.); mmartine@ugr.es (M.M.-B.); 2 Faculty of Science, University of South Bohemia, CZ-370 05 Ceske Budejovice, Czechia; 3 Departamento de Zoología, Universidad de Granada, E-18071 Granada, Spain; mmv@ugr.es (M.M.-V.); a.gm82@hotmail.es (A.B.G.-M.); 4 Unidad Asociada Coevolución: Cucos, Hospedadores y Bacterias Simbiontes, Universidad de Granada, E-18071 Granada, Spain; jsoler@eeza.csic.es; 5 Estación Experimental de Zonas Áridas (Consejo Superior de Investigaciones Científicas, CSIC), E-04120 Almeria, Spain; angela@eeza.csic.es; 6 Instituto de Biotecnología, Universidad de Granada, E-18071 Granada, Spain

**Keywords:** bacteria, clostridia, fluorescence in situ hybridization (FISH), high-throughput sequencing, hoopoe, microbiota, mutualism, quantitative polymerase chain reaction (qPCR), uropygial gland secretion

## Abstract

The uropygial gland of hoopoe nestlings and nesting females hosts bacterial symbionts that cause changes in the characteristics of its secretion, including an increase of its antimicrobial activity. These changes occur only in nesting individuals during the breeding season, possibly associated with the high infection risk experienced during the stay in the hole-nests. However, the knowledge on hoopoes uropygial gland microbial community dynamics is quite limited and based so far on culture-dependent and molecular fingerprinting studies. In this work, we sampled wild and captive hoopoes of different sex, age, and reproductive status, and studied their microbiota using quantitative polymerase chain reaction (qPCR), fluorescence in situ hybridization (FISH) and pyrosequencing. Surprisingly, we found a complex bacterial community in all individuals (including non-nesting ones) during the breeding season. Nevertheless, dark secretions from nesting hoopoes harbored significantly higher bacterial density than white secretions from breeding males and both sexes in winter. We hypothesize that bacterial proliferation may be host-regulated in phases of high infection risk (i.e., nesting). We also highlight the importance of specific antimicrobial-producing bacteria present only in dark secretions that may be key in this defensive symbiosis. Finally, we discuss the possible role of environmental conditions in shaping the uropygial microbiota, based on differences found between wild and captive hoopoes.

## 1. Introduction

Living beings from all taxonomic groups host mutualistic microorganisms that are essential for their survival [1,2]. These microbial partners, or microbiota, often form complex communities that closely interact with their hosts. Various biological traits of the host are thus determined by both its genome and the microbiome (i.e., holobiont perspective [3]). Consequently, factors affecting the microbiomes stability and homeostasis may also have drastic consequences on the host phenotype and evolutionary history [4,5,6]. In this context, it is essential for ecological and evolutionary studies to describe the microbiota (with their key members and essential functions [7]), their dynamics throughout the host life cycle, and the possible environmental influences that affect them [5]. Such studies have been mainly performed with gut microbiota of mammals [8,9,10], but also with other microbiota with antimicrobial functions (i.e., in marine invertebrates and insects; reviewed by Flórez and collaborators [4]). However, the characterization of the microbiota and its functionality for other animal hosts (e.g., birds) in relation to their particular natural history and lifestyle still deserves more attention as it will provide essential knowledge on evolutionary biology [5].

The uropygial gland is the only avian exocrine gland isolated from the external environment, and its secretion is generally composed of fatty acids and alcohols that are used for plumage maintenance through a process called preening [11,12]. In two sister avian families, woodhoopoes (Phoeniculidae) and hoopoes (Upupidae), the secretion shows special properties (e.g., dark color and pungent odor) that are mainly considered part of a predator-deterring strategy [13]. Interestingly, in two tested avian species (i.e., red-billed woodhoopoe, *Phoeniculus purpurea*, and Eurasian hoopoe, *Upupa epops epops*, hereafter hoopoe), these unique characteristics were shown to be related to the presence of antimicrobial-producing bacteria found inside the uropygial gland [14,15,16]. The lifestyle of these species, based on the use of cavities for roosting or nesting, poses a high risk of infection and predation, and symbiotic bacteria may help preventing pathogenic diseases and repelling predators [17,18]. While woodhoopoes use cavities throughout their lives for communal roosting, hoopoes only use them for nesting [19,20]. Particularly, hoopoes use (and re-use) hole-nests that, contrary to those of most hole-nesters, are barely cleaned of feces and food debris [16]. These apparently insalubrious nest conditions could favor the colonization and growth of pathogenic microorganisms and increase infection risk for nesting hoopoes (i.e., females and nestlings) through most of the breeding period [19]. However, bacterial symbionts have been found to reduce the probability of pathogenic infection [16] because of the production of antimicrobial substances (e.g., bacteriocins from enterococci [21,22]) in nesting birds. Secretions containing antimicrobial-producing symbionts have never been found in neither reproducing males nor hoopoes out of the breeding season [16,23]; these birds produce instead a whitish secretion more similar to that of other avian species [23]. The association between hoopoes and antibiotic-producing bacteria harbored in their uropygial gland seems to be a well-established mutualistic association during reproduction. In fact, eggshells are adapted to harbor the secretion containing bacteria in special structures, which impedes trans-shell contamination of embryos [24,25]. Considering that the available evidence suggests sexual and seasonal variations in the microbiota of the uropygial gland of hoopoes, it is crucial to explore the dynamics of these bacterial communities in relation to the ecological and physiological conditions experienced during the breeding season and nesting period.

In our study, we characterize in detail the microbiota of breeding females and nestlings from captive and wild bird populations for the first time. We pay particular attention to enterococci, the group of bacteria previously described as responsible for the production of antimicrobial substances (i.e., bacteriocins). Moreover, by characterizing the microbiota of secretions of male and non-breeding female hoopoes, we pointed out variations due to sex during the breeding season. Furthermore, we studied the seasonal variation (i.e., breeding vs. non-breeding) in bacterial density of secretions of males and females. The uropygial microbiota has been described combining quantitative polymerase chain reaction (qPCR), fluorescence in situ hybridization (FISH), and pyrosequencing approaches.

## 2. Materials and Methods

### 2.1. Study Area, Study Species, and Sampling Procedures

The uropygial gland secretions collected for this study were obtained from captive and wild populations of hoopoes from southern Spain between 2009 and 2012 following the procedure previously described by Soler and collaborators [16]. Briefly, samples were collected with a micropipette and sterile pipette tips directly from the inside of the uropygial gland after separating and washing the feathers around the gland with 90% ethanol to avoid contamination. Sterile empty tubes were opened at the moment of sampling and each bird was manipulated using new latex gloves cleaned with ethanol.

Captive hoopoes were maintained in Granada (Animal facilities, Faculty of Science, University of Granada, Granada), Guadix (Field installations, University of Granada, Guadix County, Granada), and Almería (Field installations “Finca Experimental La Hoya”, Experimental Station of Arid Zones, Spanish National Research Council (CSIC), Almería). For breeding, captive breeding pairs were housed in independent cages (height × width × depth of at least 3 × 2 × 2 m) with access to soil, and were provided with live food (crickets and fly larvae) and meat (beef heart) ad libitum. Prior to pairing, in winter, captive individuals were maintained in communal cages of larger dimensions. The wild population was studied in the Hoya de Guadix (Granada), where hoopoes breed within nest-boxes (internal height × width × depth: 350 × 180 × 210 mm, bottom-to-hole height: 240 mm, hole diameter: 5.5 mm) placed in trees and buildings. Adults were marked with numbered (Spanish Ministry of Environment) and colored rings for individual identification.

At the beginning of the breeding season, the first week of March, male and female hoopoes of the captive populations were moved in pairs to the breeding cages. These cages were visited twice per week during the pre-breeding period to collect uropygial gland secretion samples and record laying dates. From early March, nest-boxes of the wild population were also visited twice per week until the first egg (i.e., laying date) was detected. Some females (i.e., non-nesting females) were not paired during the breeding season or sampled during an interbreeding period (hoopoes can lay up to three clutches during the breeding season). Breeding females (during their nesting stage) and males were sampled on day 2 after hatching of the first egg, and nestlings at 14–20 days old. The risk of pathogen acquisition and the characteristics of the uropygial secretion in nesting females are similar during the incubation and hatching periods, thus the sampling was carried out in the later to minimize the probability of nest abandonment due to our manipulation. In summary, we collected uropygial secretions from wild and captive hoopoe adults at four different stages (winter, pre-breeding, breeding, and non-nesting), and from nestlings. The number of individuals from different populations, breeding stages, sexes, and ages that were sampled to characterize the uropygial microbiota varied depending of the methodological approach used, and are described in the pertinent sections below.

Research was performed in accordance with national and regional guidelines [26]. The permits to perform this research were provided by the Department of the Environment of the Junta de Andalucía (Spain). The study was approved by the ethics committee of the University of Granada (Comité de Ética en Experimentación Animal, CEEA, Ref.: 785).

### 2.2. Fluorescence In Situ Hybridization

A small amount of uropygial secretion extracted from the gland of some individuals (20 breeding females: 4 wild and 16 captive; 20 nestlings: 8 from 6 wild nests and 12 from 5 captivity nests; and 23 captive individuals in winter: 10 males and 13 females) was used to study the abundance of metabolic active bacteria by means of fluorescence in situ hybridization. Briefly, after fixing five μL of secretion smeared on Polysine adhesion slides (Thermo Scientific, Waltham, MA, USA), cells were hybridized with Eub338 [27] labeled with cyanine 3 (Cy3) as a universal probe for bacterial RNA, and with Enc221 [28] labeled with fluorescein-5-isothiocyanate (FITC) as a probe for enterococcal RNA. Fluorescent probes were synthesized by Thermo Scientific. As positive control for the presence of bacterial DNA, we incubated smears of uropygial secretion with the DNA intercalating agent Hoechst (Sigma-Aldricht, St. Louis, MO, USA). For a detailed explanation of the sample fixation and the hybridization protocol followed, see Appendix A. Using the 100× objective of an Olympus BX51 fluorescence microscope and an Olympus DP72 camera (Olympus, Shinjuku, Tokyo, Japan), two pictures of three microscopic fields per hybridized slide were taken through the 4’,6-diamidino-2-phenylindole (DAPI, blue), fluorescein-5-isothiocyanate (FITC, green-yellow), and tetramethylrhodamine-isothiocyanate (TRITC, red) filters. As the amount of hybridized bacteria estimated with the TMARKER software [29] (version 1.20163, Nexus, Zurich, Switzerland) was repeatable within individual samples (*F*_1,78_ = 7.66, *p* < 0.001, R^2^ = 0.69 for the 39 individuals with three available microscopic fields of the same sample), the average values of bacterial density were used in the analyses. See Appendix B for detailed information of estimations of bacterial densities.

### 2.3. DNA Extraction and Purification

Total DNA for qPCR and pyrosequencing was extracted from uropygial secretion samples (2–20 μL) using the FavorPrep Genomic DNA extraction kit (Favorgen Biotech, Ping-Tung, Taiwan) according to manufacturer’s instructions, adding a lysozyme pretreatment (10 mg/mL of lysozyme at 37 °C for 30 min). Purification of PCR products during pyrosequencing library construction were performed using the GenElute PCR clean-up kit (Sigma-Aldrich) according to manufacturer’s instructions. All final elutions were made in ultrapure sterile water.

### 2.4. DNA Quantification by Quantitative Polymerase Chain Reaction

The amount of bacteria present in 2 μL aliquots of different secretion samples (captive hoopoes from winter: two females and two males; pre-breeding season: five females and five males; breeding season: five breeding females, five males, six nestlings, and three non-nesting females) was estimated by qPCR using the universal bacterial primers 338f (5′-ACTCCTACGGGAGGCAGCAG-3′) and 518r (5′-ATTACCGCGGCTGCTGG-3′) [30,31]. The standard reference included was a 197 base pair amplicon of the V3 region of the 16S rDNA produced with the same primer pair, cloned in the TOPO-TA vector (Life Technologies, Carlsbad, CA, USA), and diluted in 10-fold steps to obtain a standard curve covering concentrations from 10^10^ copies to 10^1^ copies after quantification by spectrophotometry in a Nanodrop (Thermo Scientific). Each qPCR reaction contained a 20 μL mixture including 1× SensiFAST SYBR & Fluorescein Kit mastermix (Bioline, London, UK), 0.3 μM of each primer, and 2 μL of template DNA. The qPCR was carried out in a CFX96 real-time PCR System (Bio-Rad, Hercules, CA, USA) as follows: initial denaturalization at 95 °C for 3 min, 40 cycles of 95 °C for 5 s, 55 °C for 10 s, and 72 °C for 10 s with plate read after each cycle, and a final melting curve from 55 °C to 95 °C with a temperature increment of 0.5 °C every 5 s. All quantifications were performed in duplicate and the mean values were used to approximate the number of 16S rDNA copies per microliter of the original samples with the CFX Manager Software (version 3.1, Bio-Rad). As pyrosequencing let us know the bacterial composition of some samples, the approximate number of bacteria was calculated from the 16S rDNA number of copies using the average copies per genome of the most abundant group found [32].

### 2.5. Amplicon Libraries Construction and Pyrosequencing

DNA samples for pyrosequencing were obtained during the breeding season from 48 breeding females (33 wild and 15 captive) and 22 nestlings from 22 different broods (eight wild and 14 captive). Additionally, DNA of secretions from non-nesting individuals sampled in the middle of the breeding season (April–June) was pyrosequenced. These non-nesting individuals included six breeding males (two wild and four captive) and two non-nesting, captive females (one never paired and the other one previously paired in the same season).

The V1–V3 region of the 16S rDNA was amplified with the universal bacterial primers 27F (5′-TCAGAGTTTGATCMTGGCTCAG-3′) and 533R (5′-GCTTACCGCGGCKGCTGGCACG-3′) (modified from the literature [33,34]). Those primers also included an eight-base pair barcode (specific for each sample) for multiplexing, and the pyrosequencing adaptors A and B. The PCR was performed at an annealing temperature of 52 °C, with 25 cycles of amplification, using a Phusion Flash High-Fidelity PCR Master Mix (Thermo Scientific). Then, PCR products were purified, quantified by spectrophotometry using a Nanodrop (Thermo Scientific), and pooled in equimolar concentrations. After this step, we obtained amplicons suitable to be pyrosequenced with the 454 GS FLX Titanium system (Roche, Basel, Switzerland) using the Lib-L emulsion PCR method. Pyrosequencing was performed at the Department of Genomics and Health of the Center for Advanced Research in Public Health (CSISP-FISABIO) in Valencia, Spain.

Negative controls did not yield detectable PCR amplification in 1% agarose gels during library construction, and were thus not included in the sequencing. A total of 78 samples were multiplexed for this study, from which a total of 143,412 sequences of above 320 bp were obtained after demultiplexing and quality filtering. Further filtering (see next subheading) retained 141,092 sequences, with an average sequencing depth of 1809 sequences per sample (standard deviation, *SD* = 934). The data are available under the European Nucleotide Archive (ENA) project no. PRJEB27904 (http://www.ebi.ac.uk/ena/data/view/PRJEB27904).

### 2.6. Sequence Data Analysis and Taxonomic Identification

Mothur v.1.33.3 [35] was used for sequence formatting and quantitative insights into microbial ecology (QIIME) 1.8 [36] for quality filtering, primer trimming, and demultiplexing. OTU (operational taxonomic unit) picking was based on the distribution-based clustering method with a value for abundance cutoff of 10 [37] using the October 2012 Greengenes database [38], which was trimmed to adapt it to the amplified region in our 454 dataset. Chimeric sequences were checked and filtered out using UCHIME v4.2.40 [39]. Singletons, non-bacterial (i.e., chloroplast and mitochondria) sequences, and samples with less than 400 sequences were removed from our dataset. After this step, five samples (two captive nestlings, two captive males, and one wild male) were filtered out. In order to obtain a dataset within which all samples were comparable (i.e., normalizing sequencing effort), a multiple rarefaction at 75% of the minimum number of sequences per sample (i.e., 300 out of 400) was performed, and the average OTU composition of the 10 rarefactions performed was obtained. After rarefaction, diversity indexes (see below) and core microbiota (defined here as taxa at genus level found in more than 90% of the samples within each group) were calculated using QIIME 1.8 [36]. The taxonomical classification was obtained using basic local alignment search tool (BLAST) 2.2.22 [40] with the October 2012 Greengenes database [38]. The OTUs that were not identified at genus level with this approach were taxonomically assigned using BLASTn online with the default parameters and 80% threshold identity against the prokaryotic_16S_ribosomal_RNA database of the National Center for Biotechnology Information (NCBI). Three samples from captive individuals (one female and two nestlings) showed a very different taxonomic profile in their bacterial communities. This was evident already at the highest levels (class Bacilli above 45%, and for two of the samples, also class Gammaproteobacteria above 35%; these two groups are rarely present in the rest of the samples, and always below 15%; see Figure S1), thus they were discarded from statistical analyses as outliers.

### 2.7. Statistical Analyses

Bacterial densities estimated from qPCR were used to test differences between sexes and among different reproductive phases, along with their interaction, using a two-way analysis of variance (ANOVA) with sex and phase as the independent factors. In addition, Tukey multiple comparisons test was used for post-hoc comparisons. Variance within groups of samples was homogeneous (Levene’s Test; sex–age: *F*_2,27_ = 1.18, *p* = 0.323; reproductive phase (winter, pre-breeding, and breeding): *F*_3,29_ = 1.74, *p* = 0.181), which validates the use of the ANOVA [41]. Bacterial densities estimated from FISH were used to test differences between breeding females and nestling secretions also using ANOVA (Kolmogorov–Smirnov goodness-of-fit test, *p* > 0.2).

Pyrosequencing data were analyzed using non-parametric and multivariate methods. For α-diversity analyses, the OTU richness of the samples was estimated by means of the Chao1 index [42]. The Faith’s phylogenetic diversity index [43] was used to compare samples α-diversity by means of Mann–Whitney U-test and Kruskal–Wallis test, according to the number of categories in the independent factors tested. The microbiota composition (i.e., OTUs) was compared by means of permutational multivariate analysis of variance using distance matrices (adonis [44]), based on Bray–Curtis distance matrices [45]. This approach was used to test the effects of the age (adult vs. nestling) and the living conditions (wild vs. captivity) of nesting hoopoes.

All the statistical analyses and plots were performed in the R statistical environment version 3.4.4 [46], using the packages “stats” (one-way and two-way ANOVA, U-test, Kruskal–Wallis test) and “graphics” (box plots). Additional functions from the “mulcomp” [47] and “ggplot2” [48] packages were used to perform the Tukey multiple comparisons post-hoc test and to visualize the interaction plots, respectively. Community composition (i.e., β-diversity) analyses based on Bray–Curtis distances [45] were performed using the adonis function of “vegan” R package [44]. Plots for Principal Coordinate Analisys (PCoA) were obtained using EMPeror [49] as implemented in QIIME 1.8 [36].

## 3. Results

### 3.1. The Uropygial Microbiota during the Breeding Season

**Figure 1:**
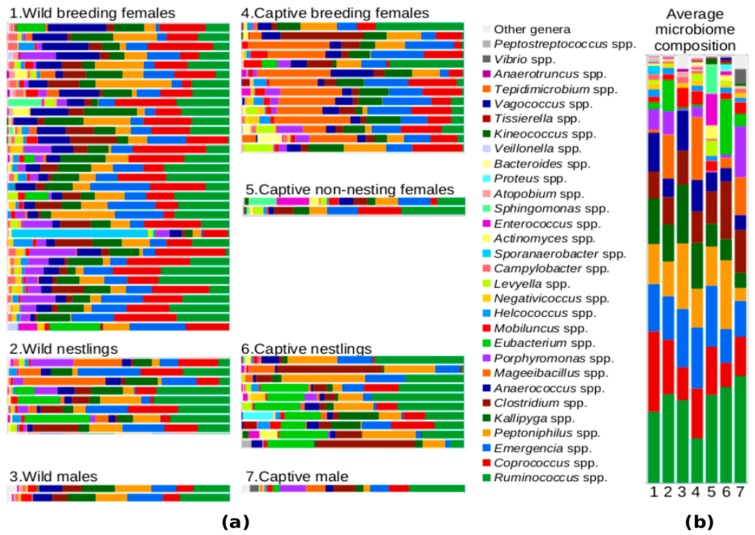
Relative abundance of genera in the hoopoe uropygial microbiota during the breeding season. Samples are organized in groups according to living conditions and sex and breeding phase. Both individual samples (**a**) and average composition for each group (**b**) are shown.

The uropygial microbiota consisted of 621 OTUs distributed within 68 genera in four phyla according to the pyrosequencing results. On average, the bacterial communities of the uropygial secretion of hoopoes included more than 40 OTUs (Chao1 index, see Table 1) and all samples showed similar profiles even at the genus level (Figure 1). Some bacterial genera were found in both dark (breeding females and nestlings) and white (breeding males and non-paired females) secretions obtained during the breeding season (see Table S1).

Firmicutes constituted 93% of the sequences analyzed, while Proteobacteria, Bacteroidetes, and Actinobacteria were found in lower proportions (see Table 2, Figure S1). Only 14 out of the 68 bacterial genera found in our samples appeared with an overall relative abundance above 1% (12 belonging to class Clostridia, phylum Firmicutes, and the other two belonging to phyla Actinobacteria and Bacteroidetes). The prevalence of these 14 genera was relatively high; 12 were present in 50% of the samples, and eight in 90% of them, including individuals of all groups (see Table S1). One genus (*Peptoniphilus*) showed 100% prevalence in our samples. The prevalence of three additional genera (one Proteobacteria and two Actinobacteria) showing low relative abundance (i.e., <1%) was also high (>50% of the samples, see Table S1).

The core microbiota included nine genera out of 57 (three present in all samples) for breeding females and seven genera out of 44 (six present in all samples) for nestlings. All the genera found in the nestling core microbiota were also found in that of breeding females. For males and non-nesting females during the breeding season, the core microbiota consisted of 13 genera out of 36 and 19 genera out of 27, respectively. Dark secretions from nesting individuals showed 28 genera (including possible antimicrobial producers: *Vagococcus* and *Parascardovia*) that were not found in white secretions (from males and non-nesting females), while three genera were found only in the latter. Two of these genera were found only in male secretions and one in non-nesting female secretions. The summary of the number of OTUs included within each genus, their overall abundance and their prevalence within groups of samples are shown in Table S1.

**Figure 2:**
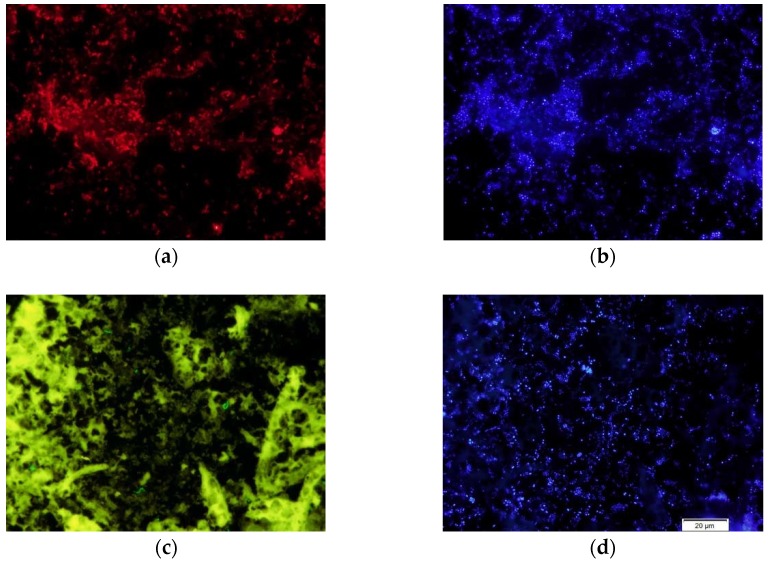
Fluorescence in situ hybridization (FISH) of the uropygial secretion. Fluorescence microphotographs of a hoopoe breeding female secretion hybridized with the universal probe Eub338 labeled with cyanine 3 (Cy3, red) (**a**) and the genus *Enterococcus*-specific RNA probe Enc221 labeled with fluorescein-5-isothiocyanate (FITC, green-yellow) (**c**). Images (**b**,**d**) are from the same microscopic fields as (**a**,**c**), respectively, taken through the 4′,6-diamidino-2-phenylindole (DAPI, blue) filter to observe the staining of bacterial DNA with Hoechst.

Enterococci were also detected by pyrosequencing in 47% of the uropygial samples (*N* = 70), although in low abundance (mean (*SD*) = 0.65% (2.14), median = 0.00%) (see Table S1). This result is similar to the one obtained when applying FISH to smears of secretions (Figure 2). Enterococci were detected in 55% of breeding females samples (*N* = 20), and in 35% of nestlings samples (*N* = 20). In any case, these bacteria constituted a very low proportion of the bacterial cells stained with the Eub338 probe (females: *N* = 20, mean (*SD*) = 0.0012% (0.0025); nestlings: *N* = 20, mean (*SD*) = 0.0011% (0.0032)), even when considering only samples with detected enterococci (females: *N* = 11, mean (*SD*) = 0.0021% (0.0031); nestlings: *N* = 7, mean (*SD*) = 0.0031% (0.0035)).

### 3.2. Seasonal and Sexual Differences in Bacterial Densities

**Figure 3:**
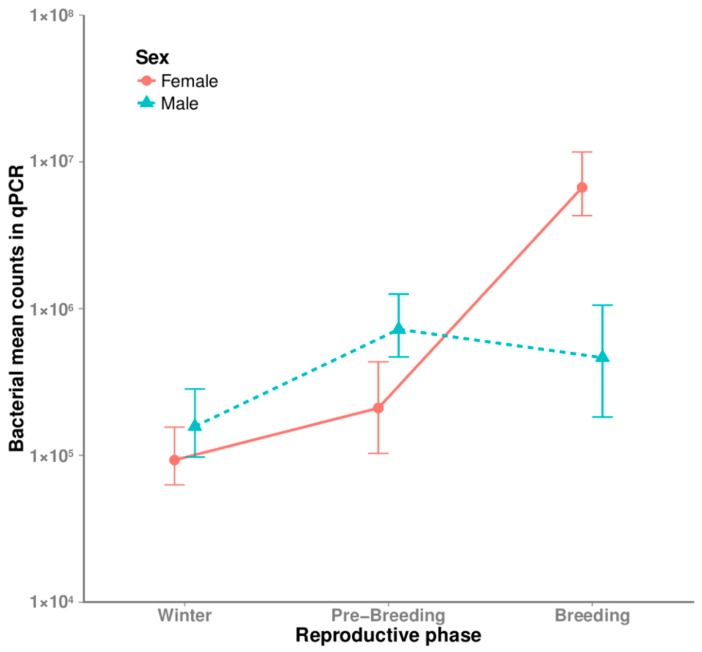
Interaction plot showing quantitative polymerase chain reaction (qPCR) results for bacterial counts per microliter of uropygial secretion. Bacterial density mean and standard deviation are represented for different reproductive phases of females (winter *N* = 2, pre-breeding *N* = 5, breeding *N* = 5) and males (winter *N* = 2, pre-breeding *N* = 5, breeding *N* = 5).

There was a significant interaction of sex and reproductive phase effects in the qPCR total bacterial densities (two-way ANOVA with interaction; *F*_2,18_ = 4.46, *p* = 0.027), because male samples (always white) showed lower bacterial loads in the three phases, while female samples increased from lower loads in the winter phase (white secretion) to higher loads in the breeding phase (dark secretion) (post-hoc, Tukey; T = 3.68, *p* = 0.017 for sexual differences during the breeding phase, and T = 3.83, *p* = 0.013 for differences among reproductive phases in females) (see Figure 3).

The qPCR total bacteria densities of female secretions during the breeding season did not depend on their breeding status (Mann–Whitney U-test; *U* = 13.0, *p* = 0.143; breeding females samples: *N* = 5, mean (*SD*) = 6.70 × 10^6^ (5.61 × 10^6^); non-nesting females samples: *N* = 3, mean (*SD*) = 2.09 × 10^6^ (1.88 × 10^6^)).

**Figure 4:**
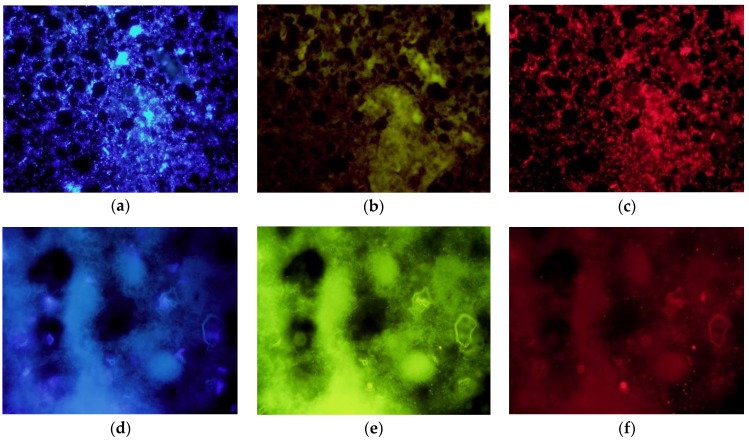
Fluorescence in situ hybridization of dark and white uropygial secretions. Fluorescence microphotographs of the dark uropygial secretion of a hoopoe breeding female (**a**–**c**), and a white secretion of a female in winter (**d**–**f**) hybridized with the universal probe Eub338 marked with Cy3 to stain bacterial RNA, and with Hoechst to stain DNA. The three images in each line are of the same microscope field, but are taken with the DAPI (blue), fluorescein-5-isothiocyanate (FITC, green-yellow), and tetramethylrhodamine-isothiocyanate (TRITC, red) filters. In the first sample, bacteria are stained in blue (**a**) and red (**c**). The images taken with the green FITC filter are included to show that a fine grain texture usually found in white secretions (example in **d**–**f**) does not correspond to stained nucleic acids because they show the same aspect in the three filters.

White and dark secretions also showed very different aspects when observed under fluorescence microscopy (Figure 4). While all smears of dark secretions from nesting individuals (i.e., breeding females and nestlings) were full of bacteria stained with both Hoechst and the Eub338 probe (an example in Figure 4a–c), white ones sampled in winter appeared without bacteria in all except one sample from females (females: *N* = 13; males: *N* = 10) (Figure 4d–f).

### 3.3. Age and Environmental Effects in the Hoopoe Uropygial Microbiota of Nesting Individuals

Dark secretions of breeding females and nestlings did not differ in the amount of bacteria stained with Hoechst per microscope field (one-way ANOVA; *F*_1,38_ = 1.30, *p* = 0.261; females: *N* = 20, mean (*SD*) = 17,418.8 (8,270.4); nestlings: *N* = 20, mean (*SD*) = 21,051.8 (11,600.3)). The same results were obtained when considering the proportion of metabolically active bacteria (i.e., stained with the Eub338 probe) (one-way ANOVA; *F*_1,38_ = 3.60, *p* = 0.808; females: *N* = 20, mean (*SD*) = 84.1% (33.9); nestlings: *N* = 20, mean (*SD*) = 61.1% (42.3)). When the total bacteria densities were assessed by qPCR, dark secretion samples from nestlings (*N* = 6, mean (*SD*) = 1.90 × 10^6^ (2.39 × 10^6^)) tended to contain less bacteria than those of breeding females (*N* = 5, mean (*SD*) = 6.70 × 10^6^ (5.61 × 10^6^)) (*U* = 26.0, *p* = 0.052). The results from the pyrosequenced microbiota showed that the bacterial α-diversity of secretions of breeding females did not significantly differ from those of nestlings (*U* = 513, *p* = 0.19). However, when comparing the β-diversity of breeding females and nestlings, differences were statistically significant (adonis; R^2^ = 0.035, *p* < 0.001) (see Figure S2A).

**Figure 5:**
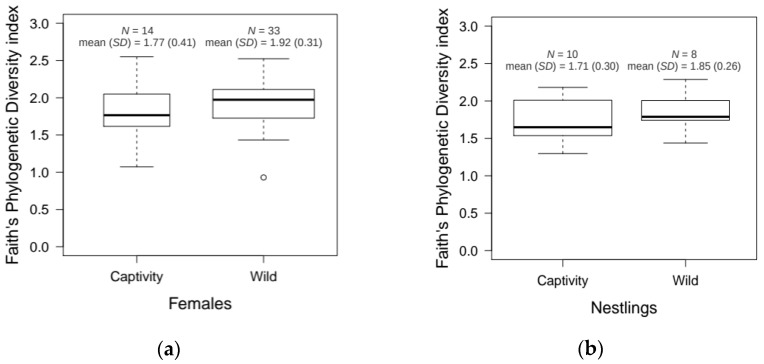
α-Diversity of hoopoes uropygial microbiota in wild and captivity populations. The box plots show the Faith’s phylogenetic diversity index for wild and captive breeding females (**a**), and wild and captive nestlings (**b**).

Regarding living conditions, there were no significant differences in microbiota α-diversity between samples coming from wild and captive populations, neither for breeding females (*U* = 175, *p* = 0.20), nor for nestlings (*U* = 26, *p* = 0.24). Nevertheless, wild samples showed slightly higher values for Faith’s phylogenetic diversity index than captive samples for both breeding females and nestlings (see Figure 5). On the other hand, wild and captive hoopoe uropygial secretions slightly differed in their microbiota (Figure 1). β-Diversity was significantly different between wild and captive populations for both females (adonis; R^2^ = 0.097, *p* < 0.001) and nestlings (adonis; R^2^ = 0.090, *p* = 0.042) (see Figure S3). Taking these results into account, the analyses comparing the microbiota composition of breeding females and nestlings were repeated for wild and captive populations separately. Differences were still statistically significant in the captive populations (adonis; R^2^ = 0.134, *p* < 0.001), but not in the wild (adonis; R^2^ = 0.034, *p* = 0.100) (see Figure S2B).

## 4. Discussion

### 4.1. Composition of the Uropygial Microbiota: Clostridia as Key Partners

The present work broadens our knowledge on microbiota and its dynamics, describing for the first time the taxonomic composition of the microbiota associated with the hoopoe uropygial gland in different life stages. Altogether, the uropygial microbiota consisted of 68 genera from four bacterial phyla. The diversity estimation in this work may be conservative given the sequencing depth obtained with the 454 platform, but should still provide a good approximation to the main taxa richness found in the uropygial secretions [50]. Although this work has a mainly descriptive nature, it opens some interesting hypotheses for future lines of research that we propose and discuss below.

The uropygial microbiota of hoopoes is dominated by Firmicutes of the class Clostridia, confirming previous results based on traditional molecular methods [51]. Bacteria in the class Clostridia are known to be essential for maintaining the stability of the gut microbiota (e.g., in humans [52]) and may play a similar role in the uropygial gland of hoopoes. They also produce butanoic acid, which is one of the most abundant volatile compounds found in hoopoe dark secretions [15]. This compound inhibits certain pathogenic bacterial strains [15], but can also function as a cell trophic factor (at least in humans) depending on its concentration [52]. In this regard, the different total bacterial (and thus Clostridia) densities found in dark and white secretions may play a role in the production of this volatile compound, which might be responsible for the increment in gland size observed in breeding females and nestlings compared with males and females out of the breeding season [23].

### 4.2. The Uropygial Microbiota of Nesting Hoopoes: Age and Environmental Contributions

The differences found between the microbiota of breeding females and nestlings support the hypothesis that some bacteria may be collected from the environment (i.e., nest of hatching) and add diversity to the existing (i.e., vertically transmitted) community (also pointed out in the literature [53,54]). As these differences are only found in the captive populations, it is possible that the captivity conditions are affecting the way the uropygial microbiota is acquired by nestlings. Additionally, in accordance with previous work [51], we have also detected differences in the microbiota of the uropygial gland of captive and wild hoopoes, which seem to increase with age. These differences also point at the influence of the environmental conditions experienced by the host in the uropygial microbial community. For example, diet is known to affect the gut microbiota of animals [55], including birds [56]. As many of the taxa detected in the hoopoe secretion are also important in gut microbiotas (i.e., Bacteroidetes and Clostridia [57]), it is possible that differences between captive and wild hoopoes were caused by the growth of some bacteria collected from the digestive tract within the uropygial gland. This hypothetical transfer of gut (e.g., fecal) bacteria could occur vertically from mothers to offspring through trans-shell contamination of embryos or by direct contact with developing nestlings [58,59], but also within individuals through behaviors like preening [60,61]. Another non-exclusive possibility explaining the detected differences in the microbiota of the uropygial gland of captive and wild hoopoes is related to the migratory behavior of wild individuals. During their migration to African winter quarters [62], wild hoopoes would become in contact with different and diverse bacteria that may be acquired and incorporated into the uropygial gland microbiota. It is also possible that hoopoes could recruit some bacteria from re-used nest materials, which would be more diverse for wild than for captive hoopoes. This last possibility is, however, unlikely because the microbiota of nest material (i.e., OTUs detected by automated ribosomal intergenic spacer-region analysis (ARISA)) did not predict the uropygial gland microbiota [63]. Further research in this sense is still needed to clarify the origin and variation of the uropygial symbiotic bacteria, as well as to explain how they are acquired and maintained through the hoopoe life cycle.

### 4.3. Hoopoe Reproductive Behavior and the Microbiota Dynamics and Functions

In previous studies using culture-dependent methods, there was no evidence of symbionts in the white secretions of non-nesting hoopoes [16], suggesting that symbiotic bacteria had to colonize the uropygial gland of breeding females every year. However, high-throughput sequencing also detected a complex microbiota in secretions of non-nesting females and males along the breeding season, and some of the bacterial genera detected in these white secretions also appeared in the dark secretions of nesting individuals (see Table S1). On the other hand, dark secretions from nesting individuals invariably showed one to two orders of magnitude higher bacterial densities than white secretions from non-nesting individuals. Thus, if the microbiota of the uropygial secretion of nesting and non-nesting individuals shares some genera, it is possible that some bacterial symbionts remain within the gland from the nestling stage and act as a baseline reservoir for the nesting phase in females. This hypothesis, however, should be further tested in future work as the low number of samples from non-nesting individuals analyzed in this study, together with the lack of information regarding microbiota composition in hoopoes out of the breeding season, does not allow us for making solid conclusions in this regard.

We also found that some potential antimicrobial-producing genera (such as *Vagococcus* and *Parascardovia*, see below) are more prevalent in dark secretions of nesting individuals than in white secretions of males and non-nesting females. This finding suggests that hosts may have evolved mechanisms to favor certain bacteria in the secretion during the phase of higher risk of pathogenic infections (i.e., nesting). Among these possible mechanisms driving the sexual variation found in the uropygial microbiota of hoopoes, we speculate with a possible role of sex hormones. Our finding of non-nesting female secretions during the breeding season with bacterial densities more similar to those of breeding females than to those of females in winter may be interpreted in accordance with this hypothesis, although the presence of similar characteristics in nestlings secretions poses a challenge to this explanation. Sexual differences have been found in the microbiota of other animals (i.e., humans [64]), and the role of sexual hormones shaping the microbiota has been experimentally tested in mammals (i.e., mice [65]). It would thus be interesting for future research to delve into these sexual differences in the hoopoe uropygial microbiota to understand the possible correlation of the symbionts and their abundance with the physiological (e.g., hormonal) status of the bird host.

The abundance of the above-mentioned antimicrobial-producing bacteria was quite low. High-throughput techniques usually do not provide good insight into rare taxa, so we may be missing other, even less abundant bacteria. However, the importance of rare taxa in microbiota functionality for hosts has been previously highlighted [50,66] and deserves further comments here. For instance, enterococci were found at low density in hoopoe uropygial secretions of less than 50% of the nesting samples using a specific FISH probe and pyrosequencing. Nevertheless, their contribution to the antimicrobial properties of the secretion has been previously reported [21,22], and their abundance on eggshells impregnated with breeding female secretion has been proven to positively impact on hatching success [24]. In the present work, we found at least two additional antimicrobial-producing candidate bacteria that appear in dark secretions and could have a defensive function: *Vagococcus* and *Parascardovia*. Bacteria in the genus *Parascardovia* (related to the genus *Bifidobacterium*) are acetic acid and lactic acid producers [67], both compounds with known antimicrobial properties [68]. The genus *Vagococcus* is phylogenetically related to the genera *Enterococcus* and *Carnobacterium*, and it is widely distributed in nature. Some strains of *Vagococcus* have been found to produce bacteriocins active against *Listeria*, *Staphylococcus*, and *Hafnia* [69]. Further studies isolating and characterizing these bacteria and their antimicrobial products, as well as analyzing their covariation with other microbiota components and with the life cycle of the bird host, will contribute to shed light on this unique symbiotic interaction between hoopoes and uropygial bacteria.

## 5. Conclusions

We have found a characteristic bacterial assemblage in the hoopoe uropygial microbiota, which varies depending on environmental conditions and life cycle of the host. Particularly, we found interesting microbiota dynamics (mainly symbiotic bacteria proliferation) in association with reproduction. Maximal bacterial densities occur during nesting, the stage with the highest risk of bacterial infection. During this phase, antibiotic-producing bacteria in the uropygial microbiota are key for hoopoes. However, because of the limited sample sizes for some of the analyzed factors, these conclusions should be considered cautiously and further tested using larger sample sizes. Future work should also delve into the mechanisms of acquisition of the main hypothesized functional taxa, as well as into determining the roles of the different components of the core uropygial gland microbiota. Another important line of research should focus on the dynamics of the uropygial microbiota in the specific context (e.g., reproduction, environmental conditions) of the hoopoe host.

## Figures and Tables

**Table 1:** α-Diversity indexes (Chao1 index and Faith’s phylogenetic diversity index) of the uropygial microbiota along the breeding season. Average and standard deviation are shown for different hoopoe groups (breeding females, nestlings, males and non-nesting females).

	*N*	Chao1 Index	Faith’s Phylogenetic Diversity Index
**Breeding females**	47	47.9 ± 9.6	1.88 ± 0.34
**Nestlings**	18	43.0 ± 8.2	1.77 ± 0.28
**Breeding males**	3	59.3 ± 9.0	3.11 ± 0.15
**Non-nesting females**	2	50.0 ± 7.1	1.97 ± 0.39

**Table 2:** Average bacterial composition at class level (in proportion of sequences) of hoopoe uropygial microbiota for breeding females, nestlings, males, and non-nesting females. The average for the total sampled hoopoe population is given at phylum level.

Phylum; Class	Breeding Females	Nestlings	Males	Non-Nesting Females	Total
Actinobacteria; Actinobacteria	0.002	0.002	0.003	0.015	0.006
Bacteroidetes; Bacteroidia	0.046	0.029	0.042	0.002	0.030
Firmicutes; Bacilli	0.004	0.011	0.011	0.074	0.926
Firmicutes; Clostridia	0.931	0.945	0.896	0.832	
Proteobacteria; Alphaproteobacteria	0.004	0.000	0.003	0.068	0.038
Proteobacteria; Betaproteobacteria	0.000	0.000	0.007	0.000	
Proteobacteria; Epsilonproteobacteria	0.011	0.003	0.013	0.005	
Proteobacteria; Gammaproteobacteria	0.001	0.009	0.025	0.002	

## Supplementary Materials

The following are available online at http://www.mdpi.com/2073-4425/9/8/407/s1, Figure S1: Individual microbiota taxonomic profile at class level. Figure S2: PCoA plots of nesting hoopoes according to their age. Figure S3: PCoA plots of nesting hoopoes according to their living conditions. Table S1: Summary of relative abundance and prevalence of the genera found in different samples of hoopoe sequenced in this study.

## Appendix A

### Fluorescence In Situ Hybridization: Hybridization and DNA Labeling with Hoechst

A small amount of the uropygial secretion extracted from the glands of some individuals (20 breeding females: 16 captive and 4 wild; 20 nestlings: 12 from 5 captivity nests and 8 from 6 wild nests; and 23 captive individuals in winter: 10 males and 13 females) was used to study abundance of metabolic active bacteria by means of fluorescence in situ hybridization. In the case of dark secretions (breeding females and nestlings), two smears of 5 μL for each individual were prepared in two different Polysine glass slides (Thermo Scientific, Waltham, MA, USA) by extending the drop to form a layer as thin as possible with the help of a second clean slide rubbed over the secretion. One of the slides was used to estimate the total amount of bacteria and the other to estimate the amount of enterococci. In the case of white secretions (winter individuals), all the available volume of secretion for each individual was used to prepare a single smear to study total amount of bacteria in the same way as with dark secretions. Smears were immediately fixed in 4% paraformaldehyde; washed in sterile distilled water; dehydrated in successive 50%, 80%, and 96% ethanol baths; air-dried; and stored in darkness at 4 °C until hybridization.

Smears were hybridized several months after collection. To permeate cell walls, 10 μL of lysozyme solution (100 mM Tris hydrochloride (Tris-HCl); 50 mM ethylenediaminetetraacetic acid (EDTA); pH 8; 2 mg/mL lysozyme, USB Corporation, Cleveland, OH, USA) were added to each smear and incubated at 37 °C during 60 min in a wet atmosphere. Then, slides were washed with filtered distilled water; air-dried; and dehydrated in successive 50%, 80%, and 96% ethanol baths.

Pre-hybridization was performed by dropping 5 μL of Dig Easy Hib detergent (Roche, Basel, Switzerland) and incubating slides at 45 °C during 2 h in a wet atmosphere. To wash the detergent, slides were immersed in pre-hybridization buffer (20 mM Tris-HCl; 0.9 M NaCl; sodium dodecyl sulfate (SDS) 0.01%; pH 7.2–7.4) and in filtered distilled water, and then slides were dried at 28 °C.

While the universal probe Eub338 (5’-GCTGCCTCCCGTAGGAGT-3′ [27]) labeled with Cy3 (Biomers.net, Ulm, Germany) was used for all bacteria identification and detection, the probe Enc221 (5′-CACCGCGGGTCCATCCATCA-3′ [28]) labeled with FITC (Biomers.net) was used for specific identification of enterococci. Hybridization was performed by adding 30 μL of the probe-containing hybridization buffer (1 μL probe; 20 mM Tris-HCl; 0.9 M NaCl; SDS 0.1%; formamide 25%, Sigma-Aldrich; pH 7.2–7.4) to the smears and incubating at 45 °C overnight in a wet atmosphere. Non-especific hybridization was controlled by increasing the temperature to 48 °C during 20 min in a wet atmosphere. Non-hybridized probes were washed away by immersing the slides successively in hybridization buffer and distilled water during 2 min. Slides were dried at room temperature. Hybridization buffers were pre-heated to ensure that SDS was dissolved in such high NaCl concentration. Afterwards, buffers were kept at room temperature.

After hybridization, 10 μL Hoechst (10 μg/mL, Sigma-Aldricht, St. Louis, MO, USA) were added to smears and the mixture was maintained for 30 min in a wet atmosphere to stain DNA as positive control for the presence of bacteria. Then, slides were washed with phosphate-buffered saline (PBS) for 2 min and dried at room temperature.

Slides were mounted applying a drop of antifading solution (Vectashield Mounting Medium H-1000, Vector Laboratories, Burlingame, CA, USA) and a coverslip, and then sealed with varnish.

The whole process was performed maintaining slides in the dark to avoid fading of the fluorescent probes and Hoechst dye, and they were stored in dark boxes at 4 °C until visualization under fluorescence microscopy.

## Appendix B

### Fluorescence In Situ Hybridization: Quantification

Slides were observed through an Olympus BX51 fluorescence microscope at 100× magnification with DAPI (blue), FITC (green-yellow), and TRITC (red) filters. Three different fields per slide with a homogeneous distribution of secretion were photographed. For each of these fields, two pictures were taken: one with the DAPI filter (for Hoechst stain) and another one with the adequate filter for each probe (Enc221-FITC was detected with FITC filter and Eub338-Cy3 with TRITC filter). Pictures were captured using the 100× objective and an Olympus DP72 camera, and processed by the cellSens 1.4.1 software (Olympus, Shinjuku, Tokyo, Japan). The amount of bacteria (see quantification method below) detected with Hoechst was repeatable within individuals (*F*_1,78_ = 7.66, *p* < 0.001, R^2^ = 0.69 for the 39 individuals with three available microscopic fields of the same sample). Therefore, we used the mean of the number of bacteria obtained for the three pictures with each filter as the count value of each individual for that filter.

The amount of bacteria stained with Hoescht and Eub338-Cy3 was estimated with the TMARKER v1.20163 software [29]. For this, we trained the software to recognize positive and negative superpixels with the semiautomatic labeling application using at least 150 positives and 150 negatives of variable intensity manually selected on a typical picture of a sample. Superpixels were created by the software based on the color histogram of images, with our specification of the number of superpixels, and with the maximum smoothness value. The number of superpixels to be created was decided by examining which of several values best resulted in a grid with the size of the stained cells. This was 70,000 for the blue stained DNA of cells in Hoechst pictures and 45,000 for the red stained RNA of cells in Eub338-Cy3 pictures. The classifier trained for each probe was subsequently applied to the remaining images to calculate the number of positive superpixels in each picture, and that number was adopted as an estimation of the number of stained cells.

In the case of enterococci, they were very scarce (Figure 4b), so the number of green cells visible in the images was counted manually on a computer screen. To calculate the proportion of all active cells that were enterococci in each sample, we referred the number of stained enterococci in the Enc221-FITC slide to the amount of Hoechst stained cells in that slide that were expected to be marked with the universal probe. This was given by the percentage of Hoechst stained bacteria in the Eub338-Cy3 slide that were also detected by the universal probe.
